# Emerging quality-by-design optimized HPLC method for the concurrent determination of cefixime and ornidazole: a multi-criteria green and blue environmental footprinting

**DOI:** 10.1038/s41598-026-51859-3

**Published:** 2026-05-26

**Authors:** Yasmeen E. Mostafa, Fawzi Elsebaei, Mohammed El-Sayed Metwally

**Affiliations:** Department of Pharmaceutical Analytical Chemistry, Faculty of Pharmacy, Mansoura University, P.O. Box 35516, Mansoura, Egypt

**Keywords:** Full factorial design, HPLC, Cefixime, Ornidazole, Laboratory prepared tablet, Sustainability profile, Chemistry, Environmental sciences

## Abstract

Design of experiments has emerged as an influential and proactive approach that demonstrated favored applicability in optimizing chromatographic procedures. Quality-by-design guarantees the long-standing efficiency of the developed HPLC method reducing the need for costly and resource-intensive univariate approach. A low environmental footprint, sensitive, fast, and simple HPLC method was developed for concurrent estimation of cefixime (CFX) and ornidazole (ORN) in their raw materials, laboratory-prepared tablets, and pharmaceutical formulations using 2^3^ full factorial design. The separation was performed on a cyano column through UV detection at 300 nm in less than 4 min. The elution was achieved using a mobile phase (pH = 6.0) composed of methanol and 0.3% triethylamine in ratio of (85%: 15%, v/v), respectively with a flow rate of 1 mL/min. The method’s validity was established with ICH $\:{\mathrm{Q}}_{2}{\mathrm{R}}_{1}$ regulations. Excellent linear correlation (*r* = 0.9999) was established within concentration ranges lies between 1 and 50 µg/mL and 0.5–50 µg/mL with high percent found of 100.04 ± 0.82 and 99.99 ± 1.01 for CFX and ORN, respectively. The approach exhibited high precision (% RDS < 2) and sensitivity with detection limits of 0.07 and 0.09 µg/mL and quantification limits of 0.21 and 0.28 µg/mL for CFX and ORN, respectively. Several metrics evidenced the method’s greenness and sustainability profile.

**Supplementary Information:**

The online version contains supplementary material available at 10.1038/s41598-026-51859-3.

## Introduction

From the early 1900 s, chromatography has emerged as the most used analytical technique analysis tool in many domains either in organic chemistry, pharmaceutical chemistry, and biochemistry^1^. Nevertheless, one of the great challenges in chromatography has regularly been creating a robust and trustworthy chromatographic analysis procedure. Numerous parameters that impact the analysis findings must be investigated to reach the optimum analytical conditions. However, that stage was achieved through a one-variable-at-time approach. In this traditional approach, the impact of changing one variable was examined while keeping the level of other variables constant. As a result, it has numerous drawbacks, including being laborious, time and materials-consuming, tedious and requiring large number of experiments. Additionally, neglecting the interaction between factors is considered a flaw at its core of that univariate approach^2^. Thus, there is an impressive need to overcome these disadvantages while developing a chromatographic method.

The design of experiments (DOE) system plays a crucial role in analyzing and modelling how process variables affect response variables. In other words, it is a means to assess cause and effect interactions. Statistically designed experiments produce valid and significant findings through statistical data collection and analysis^3^. DOE has been effectively implemented in various fields such as pharmaceuticals^4,5^, biology^6^, environment^7^ and food^8^. Recently, DOE has been profoundly employed to optimize the parameters of analytical techniques, such as chromatography. The fundamental objective of using DOE is to reduce time, energy, cost, and materials.

In our method, a full factorial design with two-levels (2^3^ FFD) was applied as statistically significant substitute for univariate approach to ensure high-quality analyses. The most influential factors affecting chromatographic findings were accurately determined by concurrently investigating the impact of each factor on multiple levels of the other factors. Additionally, the acquired results represent the net consequence of the full experimental domain with regards to perspective interaction between the variables leading to a more precise and reliable draw up window opposing to a one-variable-at-time approach^2^.

**Fig. 1:**
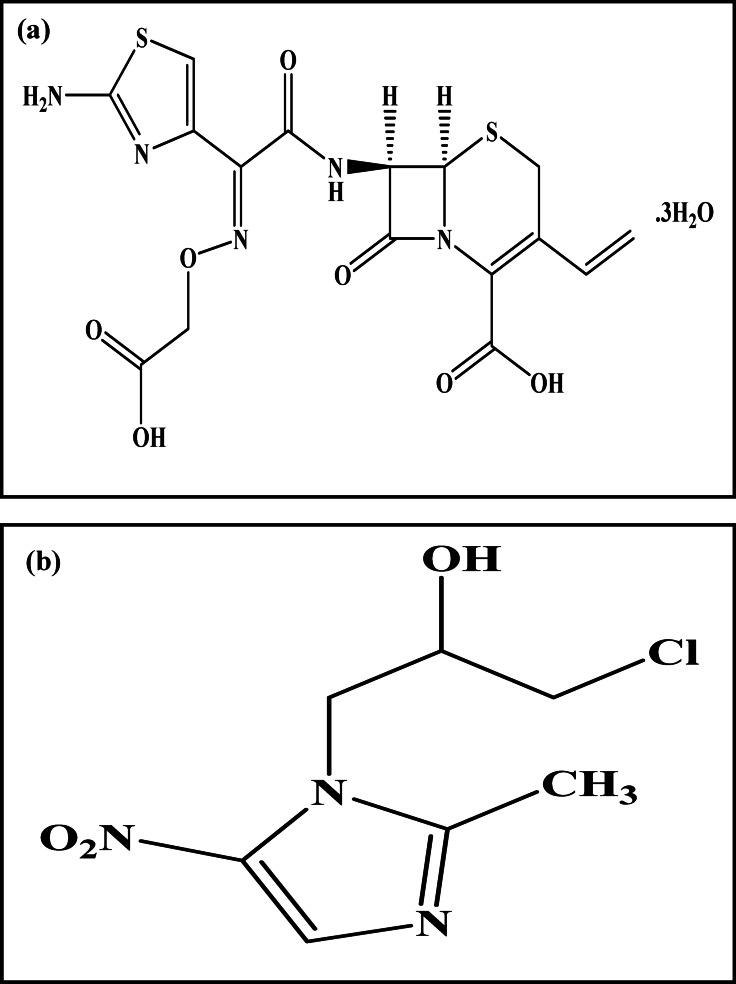
The Chemical structure of **a** cefixime and **b** ornidazole.

Cefixime (CFX) (Fig. 1a) is chemically named (6R,7R)−7-[[(Z)−2-(2-Aminothiazol-4-yl)−2-[(carboxymethoxy)imino]acetyl]amino]−3-ethenyl-8-oxo-5-thia-1-azabicyclo[4.2.0]oct-2-ene-2-carboxylic acid trihydrate^9^. CFX is an orally administered third generation cephalosporins. It is a bactericidal agent as it inhibits cell wall synthesis causing cell lysis^10^. It exhibited widespread spectrum activity towards gram positive and gram-negative bacteria^11^. It is administered to manage acute bronchitis, otitis media, uncomplicated urinary tract infections, pharyngitis/tonsillitis in addition to early and acute syphilis^12,13^. After oral intake, 40% to 50% of the drug is absorbed. About 50% is excreted unchanged in urine within 24 h^14^.

Ornidazole (ORN) (Fig. 1b.) is chemically named 1-(3-chloro-2-hydroxypropyl)−2-methyl-5-nitroimidazole. It is a nitroimidazole derivative effective against protozoa and anaerobic bacteria given orally, vaginally or via intravenous route^15^. It acts by destructing DNA causes cell death^16^. It treats many vaginal, urinary and intestinal infections such as trichomoniasis, amebiasis, and giardiasis^14,15^. It is preferred over metronidazole due to its longer half-life (12–14 h vs. 6–8 h) permits fewer daily doses and a shorter overall treatment course, improving patient compliance and convenience^15^.

Combination therapy of CFX and ORN treats bacterial and parasitic infections such as otitis media, pharyngitis, gonorrhea, urinary, gastrointestinal, and genital tract infections^17^. The literature reported various analytical methods for determining CFX and ORN either alone or combined with other drugs. The published methods for assaying CFX involving: HPLC^18–24^, spectrophotometry^23,25–28^, fluorimetry^29–31^, HPTLC^32^, voltammetry^33,34^ and capillary zone electrophoresis^35^. The published articles for assaying ORN include HPLC^36–38^, spectrophotometry^39–41^, HPTLC^42–44^, TLC^45^, voltammetry^46–48^ and capillary zone electrophoresis^49^. Besides, several methods are reported for the determination of the dual mixture including HPLC^50–53^, spectrophotometry^53–55^, HPTLC^56^. Our suggested method has an advantage over previous HPLC methods since it achieved separation of the studied drugs with higher sensitivity demonstrating low LOD and LOQ values in a faster analysis time. Moreover, our method did not rely on dangerous solvents^50–53^ or composite gradient elution^53^ or a long analysis time^52,53^. These published methods^50–53^ used acetonitrile which is less environmentally friendly and more expensive compared to methanol. Using green solvents is essential; it’s a valuable trade-off analysis for sustainable analytical practices. It is the first method to employ DOE with its abovementioned advantages in the optimization of experimental parameters in simultaneous HPLC assay of the studied drugs to provide the highest feasible analytical performance. Additionally, the unique selectivity of the cyano column toward polar compounds enabled the accurate analysis of the two drugs despite their close logP due to its moderate polarity. Moreover, the greenness of our suggested method is certified by different assessment metrics exhibiting excellent green principles.

## Experimental

### Equipment and software

The HPLC assay was conducted using a Knauer Chromatograph (Berlin, Germany) supplied with a Knauer, D-14163 injector valve. The instrument is fitted with a 20 µL loop, an Azura UVD 2.1 L UV detector, and an Azura P6.1 L pump. For pH adjustments, a pH-meter (Consort NV P-901) from Belgium was utilized through the study. The sonication was made using an USA model SS 101 H 230 ultrasonic. Membrane filters with pore size 0.45 μm (Millipore, Ireland) were utilized for filtration of mobile phase.

### Materials and reagents

Cefixime was kindly supplied from NODCAR, Cairo, Egypt. Its purity was 100.02% as quantitatively assessed by a reported method^51^. Ornidazole (Purity 99.90%) was obtained from Pharonia-Pharmaceuticals Co, Alexandria, Egypt. Ornidaz^®^ tablets (batch no. 241970 A) claimed to include 500 mg of ORN per tablet were produced by Chemipharm pharmaceutical industries, Egypt.Suprax^®^ capsules (batch no. 2055) claimed to include 200 mg of CFX per capsule were produced by Hikma pharmaceuticals industries, Egypt. Both preparations were purchased from a community pharmacy in Damietta, Egypt.

Meanwhile binary tablet dosage form was inaccessible in Egyptian medicine market marketplace; they were prepared in the laboratory. The co-formulated tablet contains CFX and ORN in a ratio of 2:5^54,57^, Consequently, this ratio was employed to prepare the tablet with excipients as legally cited^58,59^. For preparing laboratory prepared tablet, an accurate weighed quantity of the powder equivalent to 10 mg of CFX and 25 mg of ORN were thoroughly mixed with the weighed tablet additives which were 15 mg maize starch, 20 mg talc powder, along with diluent and lubricant (15 mg lactose and 7 mg magnesium stearate, respectively) for each tablet^60^.

All organic solvents used (HPLC grade), Sodium hydroxide, disodium hydrogen phosphate and triethyl amine were bought from Sigma-Aldrich, Germany. Glacial acetic acid (96%), sodium acetate, talc powder, lactose, magnesium stearate and maize starch were purchased from El-Nasr Pharmaceutical Chemicals Co., Cairo, Egypt. Orthophosphoric acid was bought from Riedelde Haën, Seelze, Germany.

### Standard stock solutions

Stock standard solutions containing 200 µg/mL from CFX and ORN were prepared separately in methanol. The stock solutions showed good stability when refrigerated for almost two weeks.

### Chromatographic conditions

The chromatographic separation was conducted on a cyano column (Hypersil™ CPS Cyano column (250 mm × 4.6 mm × 5 μm particle size). The elution was done by a mobile phase consisting of methanol and 0.3% triethylamine (TEA) in ratio of (85%: 15%, v/v), respectively. The pH was adjusted to 6.0 using 0.2 M orthophosphoric acid. The delivery of the mobile phase was at a flow rate of 1 mL/min. A membrane filter (0.45 μm) was used for filtration of the mobile phase and then sonicated for degassing. The UV detection was conducted at 300 nm. The injection volume was 20 µL. The column temperature was 25 °C.

### Procedures

#### Construction of calibration curve

Accurately measured aliquots of stock standard solutions (200 µg/mL of each drug in methanol) were transferred separately into 2 sets series of 10 mL volumetric flasks then diluted to the mark using the mobile phase to achieve the concentration range lies between (1–50) for CFX and (0.5–50) µg/mL for ORN. Under the optimum chromatographic conditions, 20 µL triplicates of each concentration were injected onto the column. Corresponding calibration curves were obtained when average peak areas were plotted versus drug concentration in (µg/mL). Corresponding regression equations were then derived.

#### Analysis of CFX and ORN in lab-synthetic mixtures

A set of lab-synthetic mixtures were obtained by transferring various aliquots of the stock solutions (200 µg/mL) of each drug into a set of 10 mL volumetric flasks within the concentration range in the pharmaceutical ratio of 2:5 for CFX and ORN, respectively. The lab-synthetic mixtures were analyzed as outlined under ‘Construction of calibration curve section’. The percentage found for each drug were mathematically estimated utilizing the derived regression equations.

#### Analysis of CFX and ORN in lab-prepared tablet

A quantity of the powder tablet containing 10 mg CFX and 25 mg ORN were accurately weighed and transferred into 100 mL volumetric flask containing 50 mL of methanol. The contents of the flask were sonicated for 45 min. The volume was completed to the mark with methanol then the mixture was filtered via Whatman no.1 filter paper. Various aliquots of the filtrate were transferred into a series of 10 mL volumetric flasks, then diluted with the mobile phase and analyzed as outlined under ‘Construction of calibration curve section’. The derived regression equations were used to estimate the nominal content of the tablet.

#### Analysis of CFX and ORN in their single marketed pharmaceutical dosage

Ten tablets of Ornidaz^®^ 500 mg were accurately weighed, ground into fine powder and blended well. Ten capsules of Suprax^®^ 200 were carefully evacuated and the content of the capsules was weighed. The average content of every separate capsule was estimated. Subsequently, the contents of all capsules were evenly mixed. The accurately calculated quantities of each drug equivalent to 20 mg were weighed and afterwards transferred to 100 mL measuring flask. About 50 ml methanol was added. Each flask mixture was subjected for sonication for 30 min, completed to the mark with methanol and then filtered. Afterwards, aliquots from the produced stock solutions containing (10.0–500.0.0.0) µg CFX and (5.0–500.0.0.0) µg ORN were transferred separately into two series of 10 mL volumetric flasks. Then, they were diluted with the mobile phase for the analysis of the studied drugs as clarified under ‘Construction of calibration curve section’. The mentioned procedure was then adopted for the assay of each drug in its marketed pharmaceutical dosage form and by following pre-constructed regression equations, the % found of each drug was calculated.

## Results and discussion

### Optimization of diverse experimental conditions

To our knowledge, optimizing HPLC methods remains a challenging and complicated process due to multiple interacting parameters. Lately, DOE provides a superior, more efficient approach. It was crucial to perform preliminary experimental trials to ensure the practicability and capability of integrating an experimental model. The preliminary experiments performed contain the following:

#### Selection of chromatographical column

Three columns were used during the experiment including Hypersil™ CPS Cyano column (250 mm X 4.6 mm X 5 μm particle size), Hypersil™ Phenyl-BDS column (250 mm X 4.6 mm X 5 μm particle size) and HyperClone™ MOS C8 column (150 mm X 4.6 mm X 5 μm particle size).

The cyano column was chosen as it produced resolved and definite sharp peaks in reasonable time of analysis. It was successfully used for the separation of the two drugs with acceptable selectivity despite their close logP.

#### Choice of suitable wavelength

**Fig. 2:**
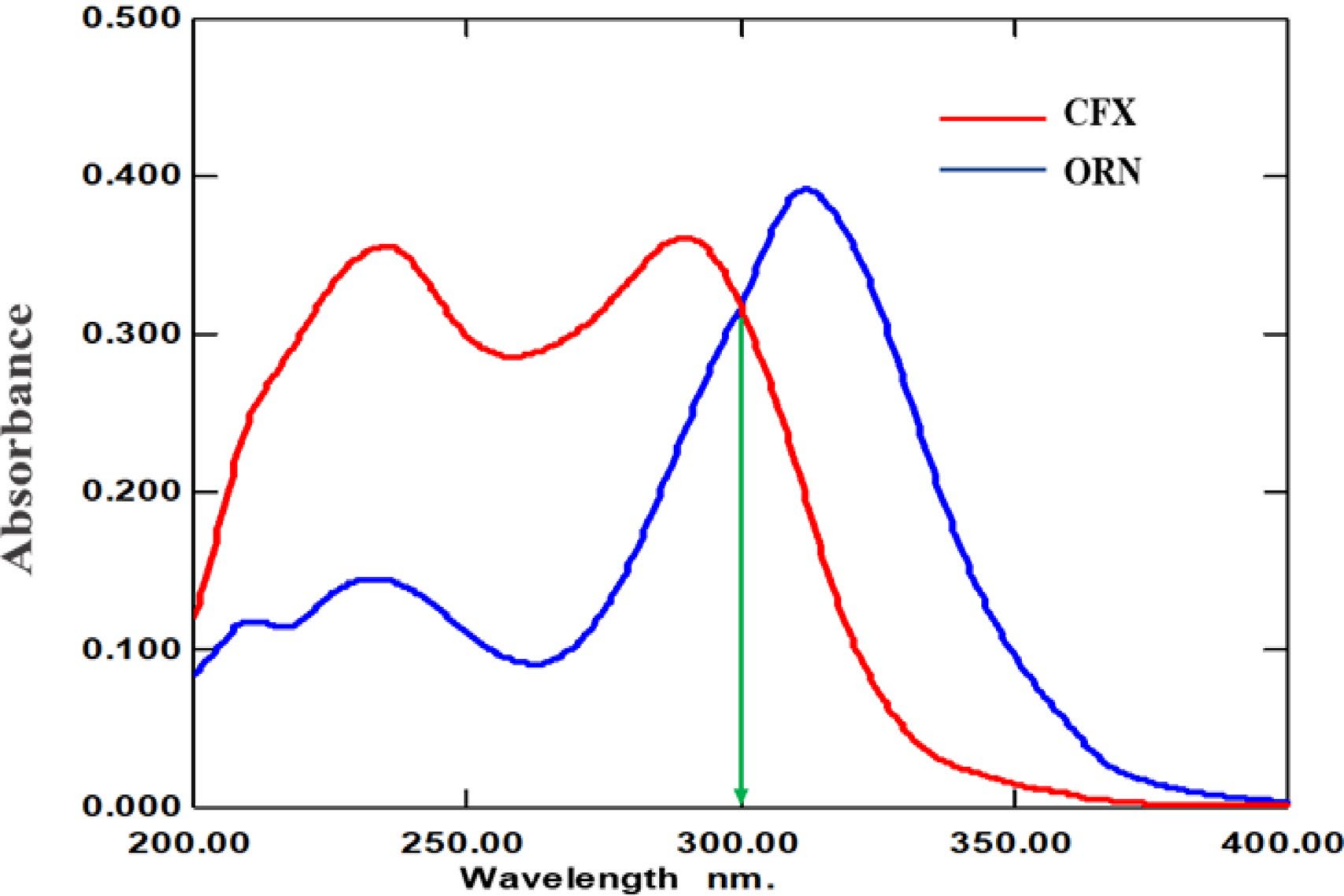
Zero-order absorption spectra of **a** 10 µg/mL CFX and **b** 10 µg/mL ORN in methanol.

The absorption spectra of both drugs were investigated, to acquire the optimum wavelength seeking for high sensitivity. Based on the UV spectrum of each drug, 300 nm was chosen for the two drugs as ideal wavelength as both drugs exhibited high absorbance at that wavelength that guarantee high sensitivity for both compounds as shown in Fig. 2.

#### Mobile phase composition

Initially, several organic solvents such as methanol, ethanol and acetonitrile were tried. Acetonitrile and methanol resulted in well-defined and resolved peaks without significant impact on separation parameters between them. Methanol was selected as the ultimate choice to avoid the major downsides of the further common solvents. Acetonitrile was excluded due to its higher significant health and environmental consequences. Moreover, it is not promptly biodegradable leading to its accumulation polluting the environment. Additionally, its price is changeably increases raising the overall cost of analytical methods. While it gave good performance, its categorization as hazardous solvent directly interferes with the principles of green chemistry making it unsuitable for green method development. Although ethanol is the greenest solvent it provided unacceptable analytical performance. So, it was excluded as it produced distorted and overlapping peaks in addition to unacceptable tailing for ORN (ORN tailing factor 3.4). Thus, choosing methanol permitted us to implement the most environmentally friendly path that meets the optimal analytical requirements of the method. Different ratios were tried, methanol ratios of 75%−85% (v/v %) were the optimal range for experimental design. Lower levels gave inadequate bad resolution, and higher levels did not significantly improve in chromatographic parameters.

The usage of ion pairing in the mobile phase aids in the resolution of analytes of similar physicochemical properties. Since the two drugs has near log P values, heptane sulphonic acid was tried as an ion pairing reagent to increase the resolution between CFX and ORN peaks. It was found that heptane sulphonic acid did not significantly affect the resolution.

Concerning the type of aqueous phase, using 0.02 M phosphate buffer of pH 5 resulted in decreasing the resolution and increasing tailing. While using 0.2 M acetate buffer of pH 6 resulted in more overlapping of the peaks and increasing tailing of both drugs. Therefore, 0.3% of TEA adjusted to pH 6 using phosphoric acid was tried and achieved the best result concerning resolution and peak tailing. Consequently, the concentration of TEA was studied. Different concentrations of TEA (0.1%−0.5%) were tried. Only concentrations of 0.1% to 0.3% were found as the optimal range for experimental design as higher concentrations did not significantly improve the results but reduce the resolution.

#### pH of the eluent

Several pH values (3–6.5.5) were prepared to examine their consequences on the resolution of peaks. This range was selected mainly to keep the physical and chemical integrity and longevity of the cyano stationary phase throughout the long-term experimental procedure. Upon decreasing pH value below 5 resulted in overlapping of the peaks, decreasing the resolution and compromising the peak shape and tailing. Best results were obtained in pH values between (5–6) so it is chosen as optimal range for experimental design.

#### Flow rate

In this study, varying flow rates in the range of 0.8 to 1.2 mL/min were examined to demonstrate its influence on the performance of the method. It was found that upon varying flow rate did not significantly improve the results. Accordingly, the best flow rate chosen during the study was 1 mL/min.

#### Column temperature

Different column temperatures were studied starting from room temperature, 30 and 40 °C. It was revealed that upon raising the temperature did not significantly improve the system suitability parameters. Consequently, the separation was conducted at room temperature (25 °C).

### Optimization of chromatographic conditions using full factorial design approach (2^3^ FFD)

FFD is deemed as a type of DOE that functions statistical models to make informative decisions based on obtaining the greatest information from the least number of experiments^61^. 2^3^ FFD was employed for optimizing the chromatographic conditions. The most significant factors affecting the performance of the method were identified. During the initial screening stage, it was concluded that there were three independent variables affecting the chromatographic separation and analytical performance of the method namely pH of the mobile phase, proportion of methanol in addition concentration of TEA (%). That could be explained that pH critically affects the ionization state of CFX and ORN, thereby affecting their polarity and retention time. While methanol (organic modifier) percentage controls the elution strength, analysis time and resolution. Concentration of TEA is essential for suppression of peak tailing and enhancing peak symmetry by minimizing residual silanol interactions. Eight Preliminary experiments were conducted as suggested by 2^3^ FFD to accomplish the best possible conditions that generated the optimum values of responses as shown in Table S1. The observed dependent factors affected by the three factors were resolution between CFX and ORN, tailing factors of CFX and ORN peaks. The capacity factor for both drugs was not affected by the three independent variables without compromising the analysis and separation of both drugs.

Afterwards, the studied drugs were assayed followed by interpretation of the obtained information. The consequential findings from each experiment were then incorporated into Minitab software to confirm the most significant dependent variables.

**Fig. 3:**
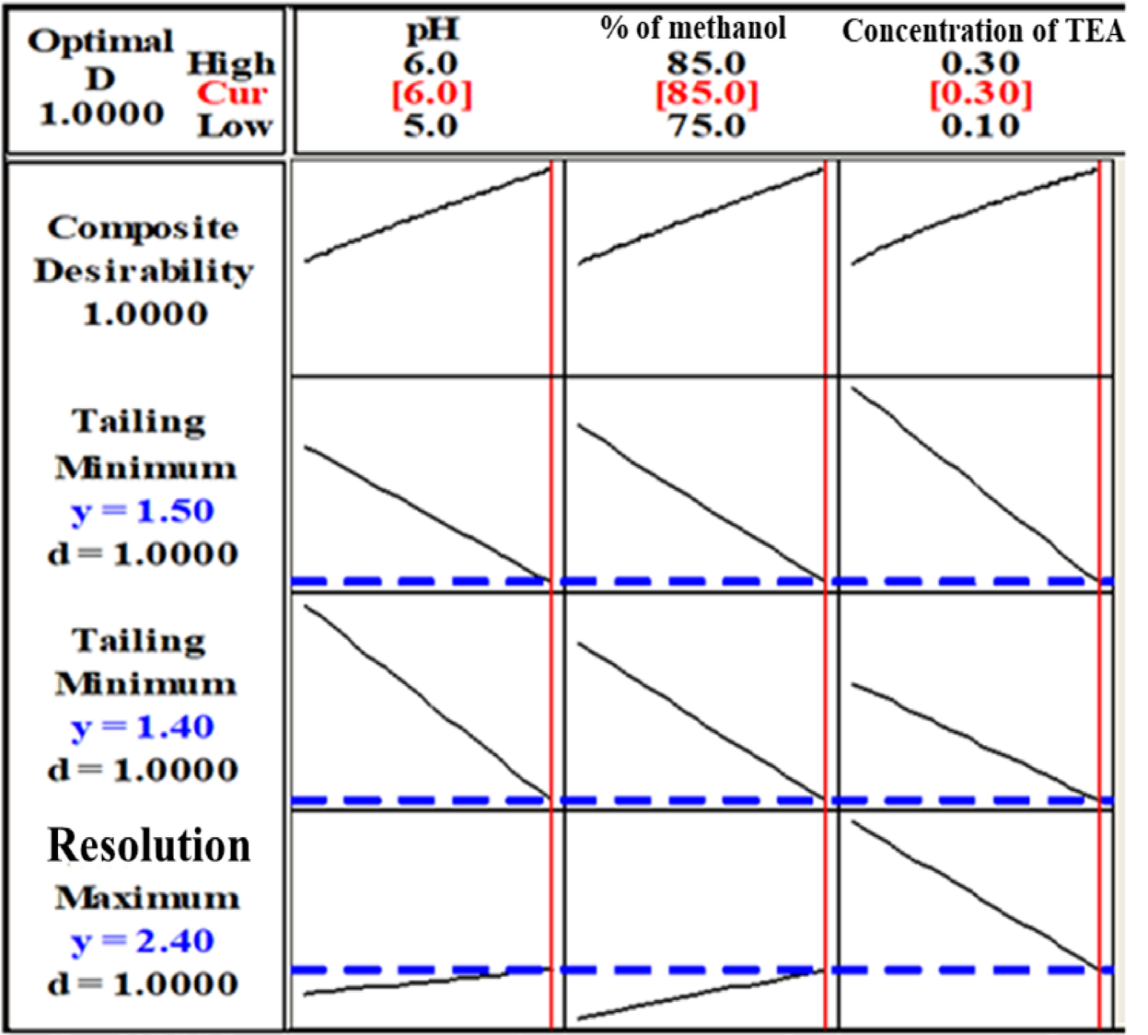
2^3^ full factorial design (FFD) optimization plot.

With the aid of Minitab, the values namely lower, target and upper for either Rs, tailing factor (CFX) and tailing factor (ORN) were determined as illustrated in Table 1. Accordingly, the optimal configuration for input values were set by response optimizer software and correspondingly, desirability values was calculated^62^. The software depends on composite desirability (D) to evaluate to what extent the responses met the satisfactory requirements for optimization. The value of composite desirability lies between zero and one. Where the closeness of this value to one is desirable rather than zero as one ensures that optimum conditions are achieved where zero indicates the obtained responses are out of their adequate limits. Minitab revealed an optimization plot and estimated the optimal solution. The optimization plot specifies the ideal experimental conditions that yield optimum responses as shown in Fig. 3. It displayed how the responses or composite desirability were influenced by each factor.

#### Factors influence resolution (Rs) between CFX and ORN peaks

The Pareto chart (Fig. S1a), the main effects plot (Fig. S2a) and the normal plot (Fig. S3a), revealed that the conc of TEA (C) most negatively affected resolution, however not statistically significant at 95% confidence. Corresponding to the interaction plots (Fig. S4a), interactions with pH and methanol at both levels (lower and higher) emphasized this negative effect. While % of methanol positively affected resolution at lower level and had little impact on resolution at its high level upon interaction with pH (Fig. S4a).

#### Factors influence tailing of CFX peak

Consistent with the Pareto chart (Fig. S1b), the main effects (Fig. S2b) and the normal plots (Fig. S3b) the conc of TEA (C) most negatively affected CFX peak tailing however not statistically significant at 95% confidence and then % of methanol and pH. According to interaction plots (Fig. S4b), the conc of TEA negatively correlated with tailing of CFX peak upon interaction with pH and % of methanol at least and higher levels. % of methanol also had a negative impact on CFX peak tailing at both levels when it interacted with pH (Fig. S4b).

#### Factors influence tailing of ORN peak

The Pareto chart (Fig. S1c), the main effects (Fig. S2c) and the normal (Fig. S3c) plots, revealed that pH (A) had the greatest negative consequence on ORN peak tailing, however not statistically significant at 95% confidence. Pareto chart (Fig. S1c) and interaction plots (Fig. S4c) showed that pH – % of methanol interaction was the most significant and % of methanol negatively affected ORN peak tailing at two levels of pH. The conc of TEA reduced ORN peak tailing upon interaction with pH and % of methanol at both levels for both factors (Fig. S4c).

Furthermore, the validity, linearity, consistency and suitability of the DOE model was proved by performing assessment between the anticipated chromatographic responses with those experimental responses. The results of regression analysis, analysis of variance, residual diagnostics and obtained R-squared values indicating good validity and reliability of the model for optimization of experimental chromatographic conditions as shown in Table S2.

### Validation criteria of the proposed method

It is noteworthy that validation is the cornerstone of any analytical procedure. Thus, the validation of the proposed method is considered in compliance with the regulations of the international council for harmonization ICH $\:{\mathrm{Q}}_{2}{\mathrm{R}}_{1}$^63^.

#### Range and linearity

The standard calibration curves for CFX and ORN were constructed by plotting the peak area against each drug concentration in µg/mL. Rectilinear responses were identified to be within the concentration range between (1–50) and (0.5–50) µg/mL for CFX and ORN, respectively. Statistical analysis of data depicted in Table 2 revealed excellent correlation coefficient values for both drugs (*r* = 0.9999) confirming good linearity of the method. The linear regression equations were derived as follows:

$$\:\mathrm{P}\mathrm{e}\mathrm{a}\mathrm{k}\:\mathrm{a}\mathrm{r}\mathrm{e}\mathrm{a}\:=\:5.2025+38.3613\mathrm{C}\;\;\;\;\;\mathrm{f}\mathrm{o}\mathrm{r}\;\mathrm{C}\mathrm{F}\mathrm{X}\:$$

$$\:\mathrm{P}\mathrm{e}\mathrm{a}\mathrm{k}\:\mathrm{a}\mathrm{r}\mathrm{e}\mathrm{a}=\:4.7258+36.4286\:\mathrm{C}\;\;\;\;\;\mathrm{f}\mathrm{o}\mathrm{r}\;\mathrm{O}\mathrm{R}\mathrm{N}\:$$

Where, C represent each drug concentration (µg/mL).

#### Limit of quantification and limit of detection

The values of LOQ and LOD were statistically determined as asserted by ICH $\:{\mathrm{Q}}_{2}{\mathrm{R}}_{1}$^63^ corresponding to the subsequent equation:

$$\:\mathrm{L}\mathrm{O}\mathrm{Q}=10{\upsigma\:}/\mathrm{S}\;\;\\\;\;\;\;\;\mathrm{L}\mathrm{O}\mathrm{D}=3.3{\upsigma\:}/\mathrm{S}$$

Considering:

*σ* is the standard deviation of the intercept.

S is the calibration curve slope. Table 2 illustrated the resulting values of LOQ and LOD evidenced the suggested method sensitivity.

#### Accuracy

**Fig. 4:**
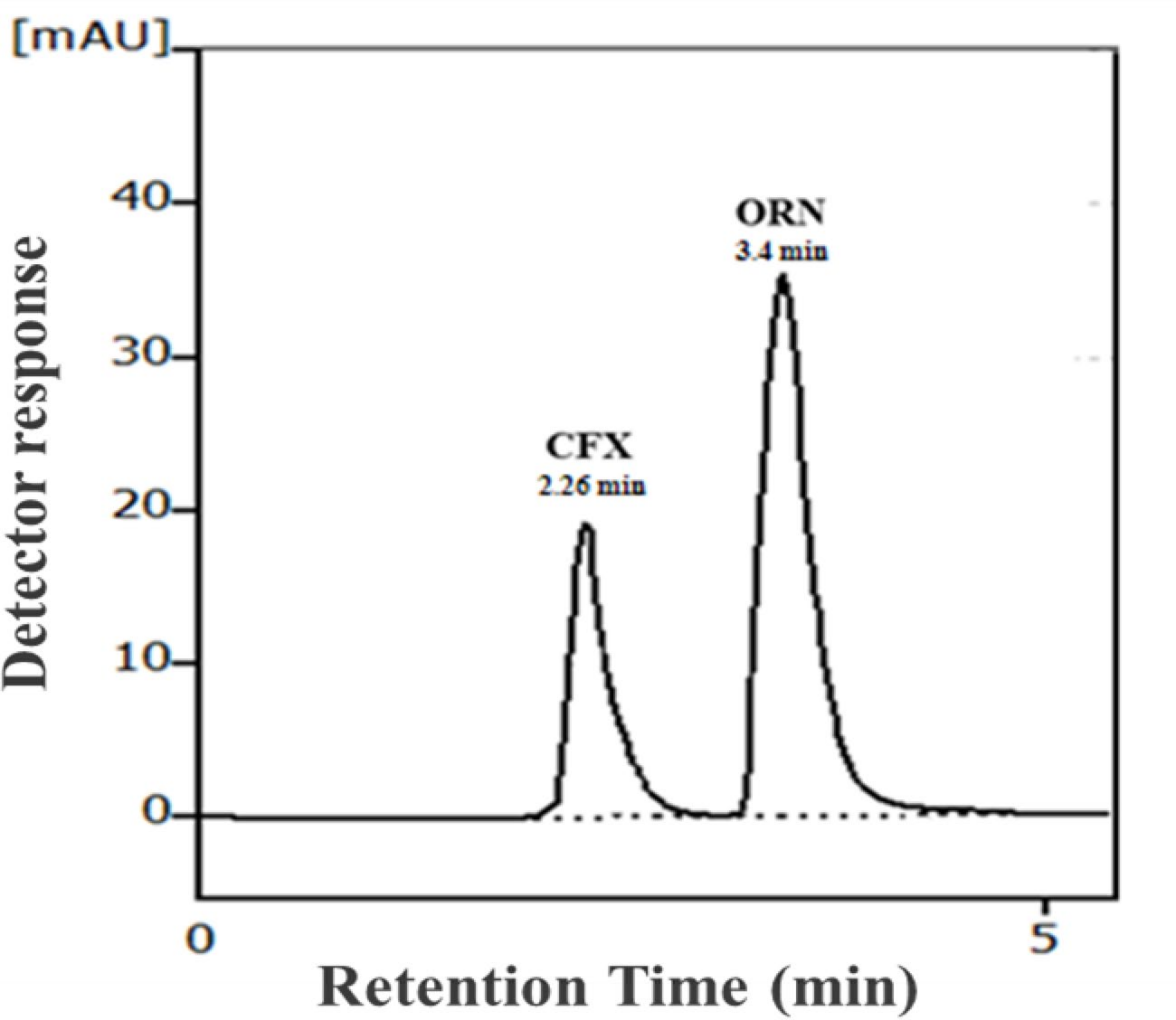
Typical chromatogram of 8 µg/mL CFX and 20 µg/mL ORN in a synthetic mixture of under the optimized chromatographic conditions.

The accuracy of the suggested method was evaluated by assaying three triplicates concentrations from each drug in raw materials and synthetic mixtures (Fig. 4) and comparing the obtained percent recoveries with those of a formerly reported reference^51^. The statistical analysis through variance ratio F-test and Student’s t- test^64^ revealed no disagreement between both suggested and reported methods concerning accuracy and precision parameters verifying the accuracy of the method as shown in Tables 3 and 4.

#### Precision

Triplicate injections of three different concentrations within the concentration range were analyzed to examine the approach’s precision. Intraday precision and inter-day precision were examined. Table 5 illustrated low obtained values of %RSD and % Error assured an acceptable precision.

#### Selectivity

**Fig. 5:**
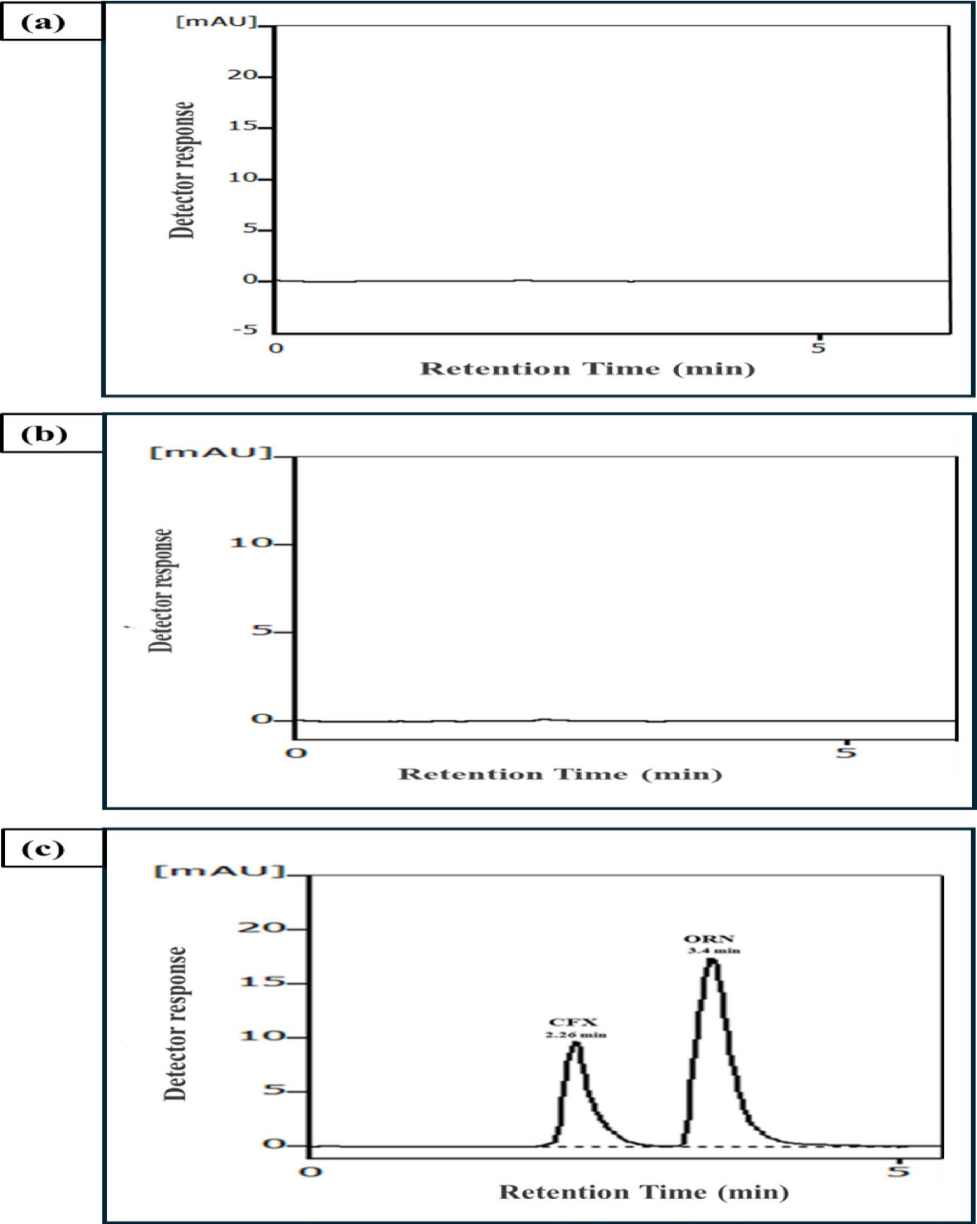
Typical chromatogram of: **a** blank, **b** placebo, **c** 4 µg/mL CFX and 10 µg/mL ORN in the laboratory prepared tablet under the optimized chromatographic conditions.

Excipients in pharmaceutical dosage forms constitute a cause of interference and may disturb the analysis of active pharmaceutical ingredients. Detection of the drugs at sensibly near UV region (300 nm) had completely inhibited any possible interaction of any combined drug matrix in their formulations. This advantage was confirmed experimentally by examining the selectivity by assaying the laboratory prepared tablets and single pharmaceutical dosage forms. The excellent percentage recoveries obtained besides low values of (%RSD) and % Error (Tables 6 and 7) confirmed that excipients did not interfere with the analysis performance as illustrated in Fig. 5a and c.

#### Robustness

The robustness of the method is inspected by intentionally changing one chromatographic parameter while keeping others unchanged. The studied variables included pH (6 ± 0.1), methanol percentage (85 ± 1, v/v %) and TEA concentration (0.3 ± 0.03). The method was sufficiently robust as illustrated in Table S3.

#### System suitability parameters

System suitability is a crucial hallmark for guaranteeing the reproducibility, optimal performance and suitability of the chromatographic system for its proposed application. Table S4 summarized system suitability parameters. Obtained values were calculated as reported in USP^65^. The cyano column was deliberately chosen to achieve the required selectivity and resolution between the two analytes. However, this selectivity was achieved at the expense of peak sharpness and overall column efficiency. The relatively low column efficiency may limit its applicability of the method for the analysis of complex matrices or the determination of low concentrations or impurities.

## The proposed method applications

### Analysis of CFX/ORN in synthetic mixtures

The established method was applied for concurrent assay of CFX/ORN in synthetic mixtures as shown in Fig. 4. Statistical assessment using Student’s t-test and variance ratio F-test^64^ summarized in Table 4 revealed that there is congruency between the findings obtained from the suggested approach and the formerly reported one^51^ in the matter of accuracy and precision.

### Analysis of CFX/ORN in laboratory prepared tablet

The studied drugs were successfully assayed in their laboratory prepared tablet by the suggested approach as shown in in Fig. 5c. The results of assay evidenced that the obtained results do not significantly differ from the comparison method^51^ by means of Student’s t-test and variance ratio F-test^64^ as abridged in Table 6.

### Analysis of CFX/ORN in their single pharmaceutical dosage forms

The proposed method was effectively implemented for the assay of CFX and ORN in their single pharmaceutical formulations. The results of the analysis shown in Table 7 concurred with those obtained from the previously reported method through variance ratio F-test and Student’s t-test^51^. Results confirmed no significant discrepancy between raw materials and the laboratory prepared tablet or single pharmaceutical formulations. This approved there is no interference from excipients.

## Evaluation of the suggested approach in aspects of sustainability and greenness

Scientific domains, in particular analytical chemistry, have undergone a rebellion in gratitude to green analytical chemistry (GAC). Remarkably, there has recently been agreement on the significance of routinely assessing the efficacy and environmental compatibility of published analytical techniques that do not severely impact both humans and the environment to guarantee ecological compatibility and sustainability.

Computerized greenness metrics conquer more concern for evaluating the greenness of analytical approaches due to their ease of application. These tools permit head-to-head assessment of methods to grasp a more environmentally friendly approach. Generally, our developed method greenness have been evaluated by varying metrics: High performance liquid chromatography environmental assessment tools (HPLC-EAT)^66^, Green certificate-Modified Eco-Scale^67^, Complementary Modified Green Analytical Procedure Index (ComplexMoGAPI)^68^ and Analytical GREEnness calculator (AGREE)^69^ and the blue applicability grade index (BAGI)^70^.

HPLC-EAT is a metric that is used to estimate the greenness of the developed HPLC method concerning the type and proportions of organic solvents utilized in sample preparation or elution in accordance with safety, health, and environmental considerations (SHE)^66^. Its highlighting outlook is to lower the amount and impact of organic solvents on the environment to cope with the GAC principles. Our suggested method gets an overall score of 5.287 as shown in Table 8 with 3.802, 0.851, 0.634 for the SHE effects, respectively.

Green certificate-Modified Eco-Scale as a metric is considered a visual upgrading of analytical Eco-Scale with a quantitative means of evaluating the greenness of the analytical approaches^67^. The calculation of the overall score is established on a penalty point system by subtracting penalty points from an ideal greenness score of 100 points. The penalty points assess analytical procedures based on occupational hazard, energy expenditure, generated waste and the quantities of used reagents and solvents. Our suggested method gets an overall score of 82 providing an excellent green method as illustrated in Table 8.

Further, ComplexMoGAPI has been recently designed for estimating the greenness of analytical techniques^68^. It is the innovative framework of ComplexGAPI with combined visual evaluation and accurate rating. Furthermore, the additional hexagonal section at the bottom offers detailed assessment of each stage in the analytical method starting from sample collection, sample preparation till the ultimate analysis of the sample. Each segment in the familiar five pictogram is assigned a color when a definite criterion is fulfilled based on their perspective influence on humans and the environment. By means of Color-implied grade from green to yellow to red, we can clearly estimate the whole ecological sustainability outline of the method in addition to a cumulative score. Our established method gets an overall score of 83, validating an excellent green method as displayed in Table 8.

Besides, AGREE provides a comprehensive evaluation of the analytical technique by allocating scores based on the 12 GAC principles. The final assessment score was the consequence of each principle’s individual score. The whole score, which ranges from 0 to 1, is displayed in the center of a visual symbolization that resembles a clock and uses color. With a total score of 0.71, our developed method was sufficiently green as displayed in Table 8.

Finally, a more innovative tool called BAGI is developed that mainly emphasize on the practicality and applicability of the developed methods in blueness aspects^70^. BAGI offers a quantitative estimation of blueness producing a pictogram on the core of ten criteria containing the category of analysis, the number of analytes, sample throughout, the instrument involved, the sample preparation, preconcentration requirements, analysis frequency, reagents and materials, automation degree and amount of sample. The assessment uses four distinct scores with equal weights, each of which contributes to the ultimate score. The detached shades of dark blue, blue, light blue, and white are designated to high, intermediate, minimal, and no conformity with the established criteria. With a score of 82.5 as displayed in Table 8, our suggested method revealed excellent practicality.

## Comparison of the method with previously published chromatographic methods

The primary novelty and significant contribution of our work did not lie in the mere act of separating two compounds, but in the advanced, systematic and quality-centric employed approach which is fundamentally absent in the cited and conventional methods. A direct comparison of our method with previously reported methods was illustrated in Table 9. Crucially, our method extends beyond “greenness” to include a “blueness” assessment which focuses on analytical practicality and efficiency. The data presented in Table 9 demonstrated the holistic green and blue advantage of our optimized method indicating a significant decrease in environmental and health impact throughout the analysis. This comprehensive sustainability profile is a novel aspect of the analytical procedure lifecycle assessment for this drug combination.

## Conclusion

The study presented announced a green, simple, sensitive, accurate, precise, and environmentally friendly HPLC method for concurrent assay of CFX and ORN in raw materials, laboratory prepared tablet and single pharmaceutical dosage forms. A cyano column has been used for such resolution extending the scope of reversed phase chromatography applicability behind regular C18 columns. 2^3^ FFD was employed for optimization of chromatographic conditions of the developed method with demonstrated excellent resolving quality in addition to minimal effort, energy, time, trial numbers and resources. As far as we are aware, it is first design of experiment-assisted method for assaying the two drugs in shorter analysis time and improved sensitivity comaring to the previous reported methods. The study provides greater simplicity, faster analysis times and more ecological options, all of which are essential for the various daily analyses carried out by quality control and pharmaceutical research laboratories. The method showed high selectivity enabling the analysis of both drugs in the laboratory prepared tablet and their single pharmaceutical formulations with satisfactory percentage recoveries without interference. Multiple metrics were employed to conduct a thorough greenness assessment of the method highlighting its high applicability and environment friendliness.

**Table 1:** Response optimization of 2^3^ full factorial design for HPLC separation of CFX/ORN mixture.

	Parameters							Optimum conditions:pH = 6.0,%methanol = 85%,concentration of TEA = 0.3%			
								Composite desirability (D) = 1			
Responses	Goal	Lower	Target	Upper	Weight	Importance		Predicted responses		Individual desirability (d)	
Rs	Maximize	1.60	2.40	3.10	1	1		2.40		1	
Tailing of CFX	Minimize	1.40	1.40	2.80	1	1		1.40		1	
Tailing of ORN	Minimize	1.50	1.50	2.20	1	1		1.50		1	

**Table 2:** Performance data of CFX and ORN by the suggested HPLC method.

Parameter	CFX	ORN
-Concentration range (µg/mL)	1–50	0.5–50
-LOD (µg/mL)	0.07	0.09
-LOQ (µg/mL)	0.21	0.28
-Correlation coefficient (r)	0.9999	0.9999
-Slope	38.3613	36.4286
-Intercept	5.2025	4.7258
-Sy/x	2.82	7.94
-Sa	1.67	4.06
-Sb	0.07	0.18
-% Error	0.31	0.36
-%RSD	0.82	1.01
-No. of Experiments.	7	8
-Mean found (%) ± SD.	100.04 ± 0.82	99.99 ± 1.01

N.B.

-S_y/x_ =standard deviation of the residuals.

-S_a_ = standard deviation of the intercept of regression line.

-S_b_ = standard deviation of the slope of regression line.

-% Error = RSD%/√ n.

**Table 3:** Application of the suggested HPLC method to the determination of CFX and ORN in their raw materials.

Compound	Proposed method			Comparison method^51^	
	Conc. taken (µg/mL)	Conc. found (µg/mL)	%Found *	Conc. taken (µg/mL)	%Found *
Cefixime	1.0	1.008	100.80	50.0	100.39
	5.0	4.935	98.70	70.0	99.50
	10.0	10.122	101.22	90.0	100.18
	15.0	14.951	99.67		
	20.0	19.950	99.75		
	30.0	30.045	100.15		
	50.0	49.990	99.98		
X^−^ ± SD			100.04 ± 0.82		100.02 ± 0.47
Student’s t test			0.05(2.31) * *		
Variance ratio (F test)			3.05(19.33) * *		
Ornidazole	0.5	0.498	99.60	125.0	100.42
	1.0	1.009	100.90	175.0	99.45
	5.0	4.956	99.12	225.0	100.20
	10.0	10.091	100.91		
	15.0	15.014	100.09		
	20.0	19.663	98.32		
	30.0	30.381	101.27		
	50.0	49.888	99.78		
X^−^ ± SD			99.99 ± 1.01		100.02 ± 0.51
Student’s t test			0.06(2.26) * *		
Variance ratio (F test)			3.92(19.35) * *		

*Average of three replicate estimations.

**Values between parentheses are the tabulated t and F values respectively, at *p* = 0.05^64^.

**Table 4:** Analytical data for the determination of CFX and ORN in their synthetic mixtures by the suggested HPLC method.

Synthetic mixture	Proposed method						Comparison method^51^	
CFX: ORN (2:5)	Conc. taken (µg mL ^**− 1**^ **)**		Conc. found (µg mL ^**− 1**^)		%Found*		%Found*	
	CFX	ORN	CFX	ORN	CFX	ORN	CFX	ORN
	4.0	10.0	4.016	10.072	100.40	100.72	100.39	100.42
	6.0	15.0	5.983	14.859	99.72	99.06	99.50	99.45
	8.0	20.0	8.098	20.066	101.23	100.33	100.18	100.20
	12.0	30.0	12.065	29.553	100.54	98.51		
X̅ ± S.D.					100.50 ± 0.54	99.66 ± 0.9	100.02 ± 0.47	100.02 ± 0.51
Student’s t test					1.30 (2.57) * *	0.72 (2.57) * *		
Variance ratio (F test)					2.07(19.16) * *	6.23 (19.16) * *		

*Average of three replicate estimations.

**Values between parentheses are the tabulated t and F values respectively, at *p* = 0.05^64^.

**Table 5:** Inter-day and intra-day precision data for CFX and ORN by the suggested HPLC method.

Compound	Concentration (µg/mL)	Intra-day precision			Inter-day precision		
		Mean ± SD	RSD (%)	Standard error	Mean ± SD	RSD (%)	Standard error
CFX	5.0	98.98 ± 0.91	0.92	0.53	98.94 ± 0.77	0.78	0.45
	10.0	99.76 ± 1.32	1.32	0.77	100.21 ± 0.99	0.99	0.57
	30.0	99.40 ± 0.70	0.70	0.41	100.50 ± 1.08	1.08	0.62
ORN	5.0	99.17 ± 1.47	1.48	0.85	100.92 ± 1.57	1.56	0.90
	10.0	98.91 ± 0.85	0.86	0.50	100.84 ± 1.09	1.09	0.63
	30.0	100.81 ± 0.84	0.83	0.48	99.47 ± 1.56	1.57	0.90

**Table 6:** Analytical data for the determination of CFX and ORN in their prepared tablet by the suggested HPLC method.

Pharmaceutical prepared tablet	Proposed method								Comparison method^51^	
(10 mg CFX: 25 ORN mg /tablet)	Conc. taken (µg mL^−1^)			Conc. found (µg mL^−1^)			%Found*		%Found*	
	CFX		ORN	CFX		ORN	CFX	ORN	CFX	ORN
	4.0		10.0	3.933		10.057	98.33	100.57	98.76	99.78
	8.0		20.0	8.042		19.830	100.53	99.15	100.11	100.42
	10.0		25.0	10.167		24.760	101.67	99.04	100.47	99.79
	12.0		30.0	11.947		29.976	99.56	99.92		
X̅ ± S.D.							100.02 ± 1.23	99.67 ± 0.62	99.78 ± 0.74	99.99 ± 0.30
Student’s t test							0.32 (2.57) * *	0.91 (2.57) * *		
Variance ratio (F test)							2.75 (19.16) * *	4.22 (19.16) * *		

* Average of three replicate estimations.

* *Values between parentheses are the tabulated t and F values respectively, at *p* = 0.05^64.^

**Table 7:** Application of the proposed method for the determination of CFX and ORN in their single pharmaceutical dosage forms by the suggested HPLC method.

Pharmaceutical Preparation		Proposed method		Comparison method^51^
	Conc. taken (µg mL^−1^)	Conc. found (µg mL^−1^)	%Found*	%Found*
Suprax ^®^ capsule (200 mg/capsule)	4.0	3.980	99.50	99.24
	8.0	8.046	100.58	100.64
	10.0	9.932	99.32	99.89
	12.0	12.080	100.67	
X̅ ± S.D.			100.02 ± 0.61	99.92 ± 0.57
Student’s t test			0.22(2.57) * *	
Variance ratio (F test)			1.16(19.16) * *	
Ornidaz^®^ tablet (500 mg/tablet)	10.0	9.870	98.70	99.23
	20.0	19.668	98.34	98.60
	25.0	24.868	99.47	99.01
	30.0	29.661	98.87	
X̅ ± S.D.			98.85 ± 0.41	98.95 ± 0.26
Student’s t test			0.39 (2.57) * *	
Variance ratio (F test)			2.43 (19.16) * *	

*Average of three replicate estimations.

* *Values between parentheses are the tabulated t and F values respectively, at *p* = 0.05^64^.

**Table 8:** The results for evaluation of the greenness of the proposed method according to HPLC-EAT, Green Certificate-Modified Eco-Scale, Complex MoGAPI, AGREE and BAGI.

1. HPLC- EAT			
2. Green certificate-Modified Eco-Scale			
Item		Penalty scores	BAGI
1. Reagent; amount			
Triethyl amine		6	
Methanol		6	
Orthophosphoric acid		2	
2. Instrument			
HPLC-UV	≤ 1.5 KW h per sample	1	
3. Occupational hazard	Analytical process hermitization	0	
4. Waste	No treatment	3	
Total penalty points		18	
Analytical Eco-Scale final score		∑ 82	
3. Complex MoGAPI		4. AGREE	5. BAGI
		

**Table 9:** The results for comparison of the proposed method with previously published chromatographic methods.

Method	Correlation coefficient		Linearity (µg/mL)		Rt (min)		LOD (µg/mL)		Experimental conditions	Greenness assessment	Blueness assessment	Ref
	CFX	ORN	CFX	ORN	CFX	ORN	CFX	ORN				
This work	0.9999	0.9999	1–50	0.5–50	2.26	3.4	0.07	0.09	cyano column with a mobile phase consisting of methanol and 0.3% TEA (85:15, v/v) UV detection at 300 nm			
Reported HPLC method	0.9992	0.9999	23–128	80–320	1.9	3.8	NA		C18 column with a mobile phase consisting of water: acetonitrile: methanol (50:25:25, v/v/v), PDA detector at 304 nm			^50^
Reported HPLC method	0.9991	0.9995	50–200	125–500	2.09	4.68	NA		C18 column with a mobile phase consisting of TEA buffer (PH 5.5) and Acetonitrile (75:25, v/v) UV detection at 295 nm			^51^
Reported HPLC method	0.9990	0.9998	60–140	150–350	4.75	8.98	0.10	0.24	C18 column with mobile phase consisting of tetra-butyl ammonium hydroxide and acetonitrile (75:25, v/v), pH = 6.5. UV detection at 254 nm			^52^
Reported HPLC method	0.9996	0.9992	1–5	1–5	10.13	13.65	NA		C18 column with gradient elution of mobile phase consisting of different proportions of 0.04 M phosphate buffer and acetonitrile UV detection at 280 nm			^53^
Reported HPLC method	0.9996	0.9993	2–10	5–25	2.1	5.6	0.16	0.33	C18 column with mobile phase consisting of methanol: water (50:50 v/v) UV detection at 298 nm			^54^

## Supplementary Information

Below is the link to the electronic supplementary material.

Supplementary Material 1

## Data Availability

The datasets generated during and/or analyzed during the study are available on reasonable request.
