# Supplementary material for: Emerging quality-by-design optimized HPLC method for the concurrent determination of cefixime and ornidazole: a multi-criteria green and blue environmental footprinting
Source: Sci Rep. 2026 May 26;16:16309. doi: 10.1038/s41598-026-51859-3 (PMC13212902; doi:10.1038/s41598-026-51859-3)
Supplement: Supplementary file 1 — Supplementary Material 1 [file 41598_2026_51859_MOESM1_ESM.docx]

**Emerging Quality-by-Design Optimized HPLC Method for the Concurrent Determination of Cefixime and Ornidazole: A Multi-Criteria Green and Blue Environmental Footprinting**

**Yasmeen E.Mostafa***, **Fawzi Elsebaei^*^**, **[Mohammed El-Sayed Metwally](https://www.sciencedirect.com/science/article/pii/S0014827X04001417#!)**

Department of Pharmaceutical Analytical Chemistry, Faculty of Pharmacy,

Mansoura University, P.O. Box 35516 Mansoura, Egypt.

* Corresponding author: Yasmeen E. Mostafa: [yasmeenesmail86@mans.edu.eg](mailto:yasmeenesmail86@mans.edu.eg), Fawzi Elsebaei: faelsepai@yahoo.com.

**Table S1. Suggested eight experimental runs to conduct 2^3^ full factorial design**

| Run number | pH | % of methanol | TEA concentration (%) |
| --- | --- | --- | --- |
| 1 | 6 | 75 | 0.3 |
| 2 | 6 | 75 | 0.1 |
| 3 | 6 | 85 | 0.3 |
| 4 | 5 | 75 | 0.1 |
| 5 | 5 | 85 | 0.1 |
| 6 | 5 | 85 | 0.3 |
| 7 | 6 | 85 | 0.1 |
| 8 | 5 | 75 | 0.3 |

**Table S2: Results of analysis of variance, R² values, and residual diagnostics for the response models.**

| **Response** | **Source** | **Degree of freedom (DF)** | **Sum of squares (SS)** | **Mean of square (MS)** | **F-value** | **P-value** | **R^2^** |
| --- | --- | --- | --- | --- | --- | --- | --- |
| **Resolution**  **(Rs)** | **Regre**ss**ion** | 3 | 1.695 | 0.565 | 12.22 | 0.018 | 90.2% |
|  | **Residual error** | 4 | 0.185 | 0.04625 |  |  |  |
|  | **Total** | 7 | 1.88 |  |  |  |  |
| **Tailing of CFX** | **Regression** | 3 | 0.85 | 0.28 | 5.40 | 0.069 | 80.2 % |
|  | **Residual error** | 4 | 0.21 | 0.0525 |  |  |  |
|  | **Total** | 7 | 1.06 |  |  |  |  |
| **Tailing of ORN** | **Regression** | 3 | 0.35375 | 0.11792 | 10.48 | 0.023 | 88.7% |
|  | **Residual error** | 4 | 0.045 | 0.01125 |  |  |  |
|  | **Total** | 7 | 0.39875 |  |  |  |  |
| **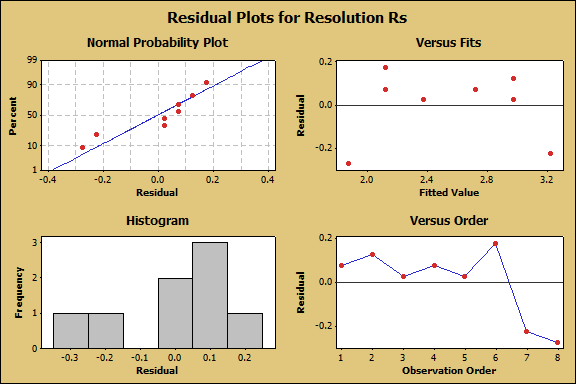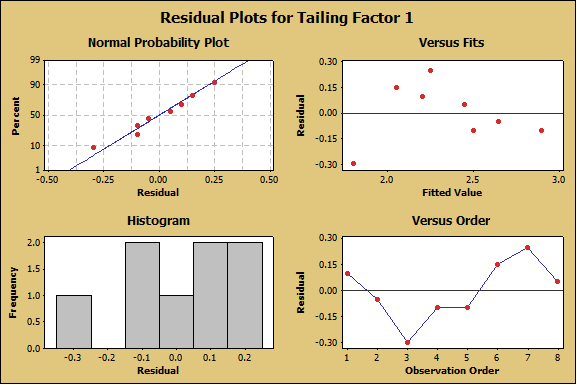Residual Diagnostics**  **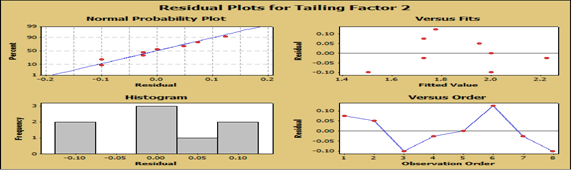** | | | | | | | |

**Table S3. Evaluation of the suggested HPLC method robustness.**

| Parameter | %Found | |
| --- | --- | --- |
|  | **CFX** | **ORN** |
| (a) pH | | |
| 5.9 | 99.80 | 99.10 |
| 6 | 98.73 | 100.17 |
| 6.1 | 99.86 | 100.45 |
| Mean± SD | 99.46 ± 0.64 | 99.91 ± 0.71 |
| %RSD | 0.64 | 0.71 |
| %Error | 0.37 | 0.41 |
| (b) Concentration of TEA (%) | | |
| 0.27 % | 98.46 | 99.57 |
| 0.3 % | 98.73 | 100.17 |
| 0.33 % | 100.58 | 98.79 |
| Mean± SD | 99.26 ± 1.15 | 99.51 ± 0.69 |
| %RSD | 1.16 | 0.70 |
| %Error | 0.67 | 0.40 |
| (C) Methanol ratio (v/v%) | | |
| 84 | 99.28 | 99.24 |
| 85 | 98.73 | 100.17 |
| 86 | 98.43 | 100.14 |
| Mean± SD | 98.81 ± 0.43 | 99.85 ± 0.53 |
| %RSD | 0.44 | 0.53 |
| %Error | 0.25 | 0.31 |

**Table S4. System suitability parameters of the suggested HPLC method.**

| Parameter | CFX | ORN |
| --- | --- | --- |
| Number of theoretical plates (NTP) | 645 | 515 |
| Selectivity | 1.70 |  |
| Resolution (R_s_) | 2.40 | |
| Retention time | 2.26 3.40 | |
| Tailing factor (T) | 1.54 | 1.57 |


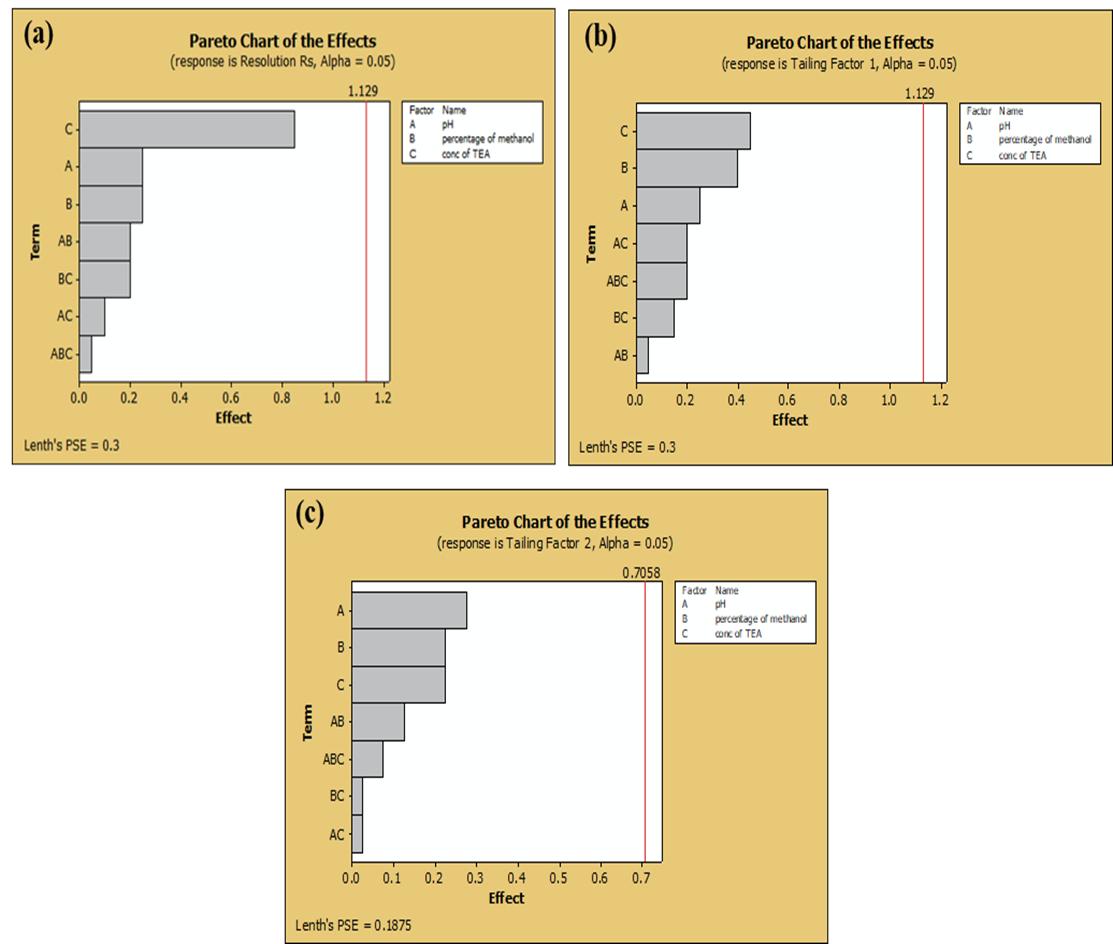


Fig. S1: 2^3^ full factorial design Pareto charts of the effects on the chromatographic responses at alpha = 0.05.


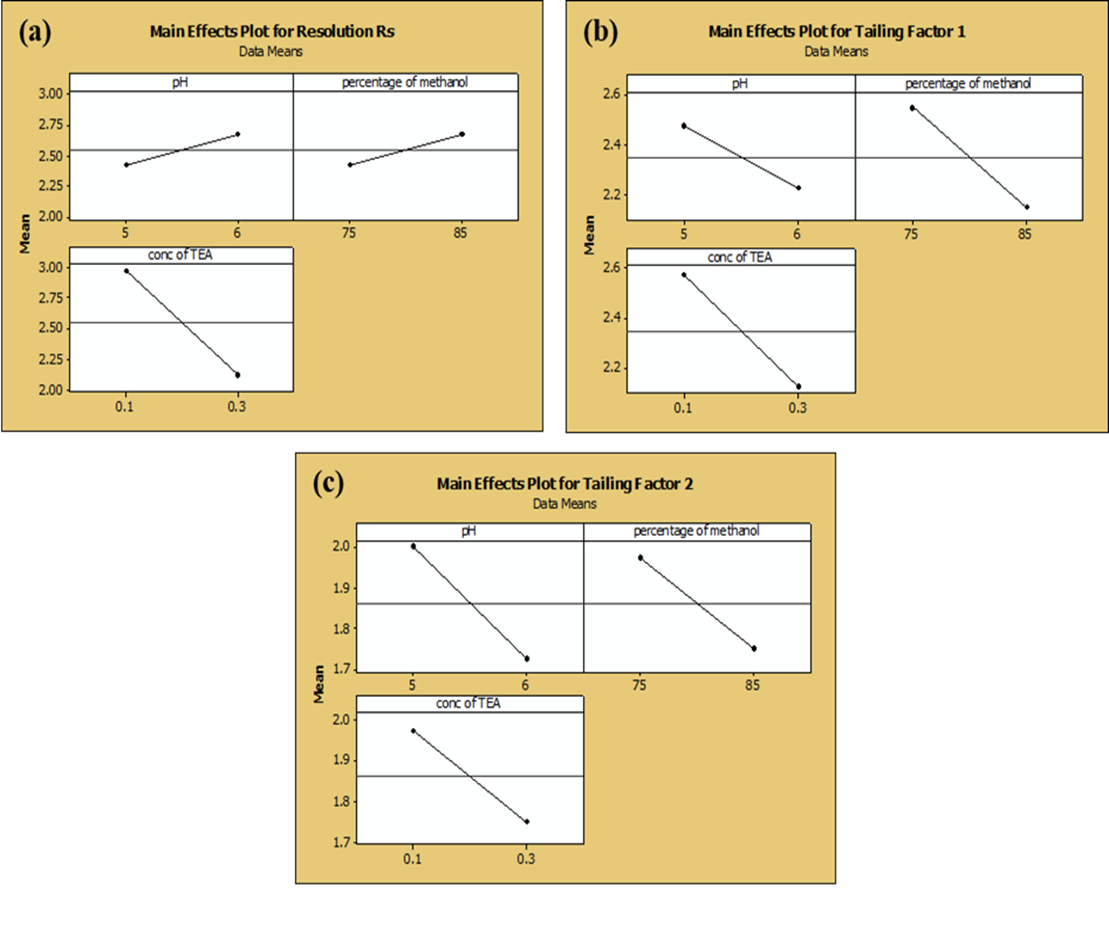


Fig. S2: 2^3^ full factorial design main effect plots for chromatographic responses by data means type.


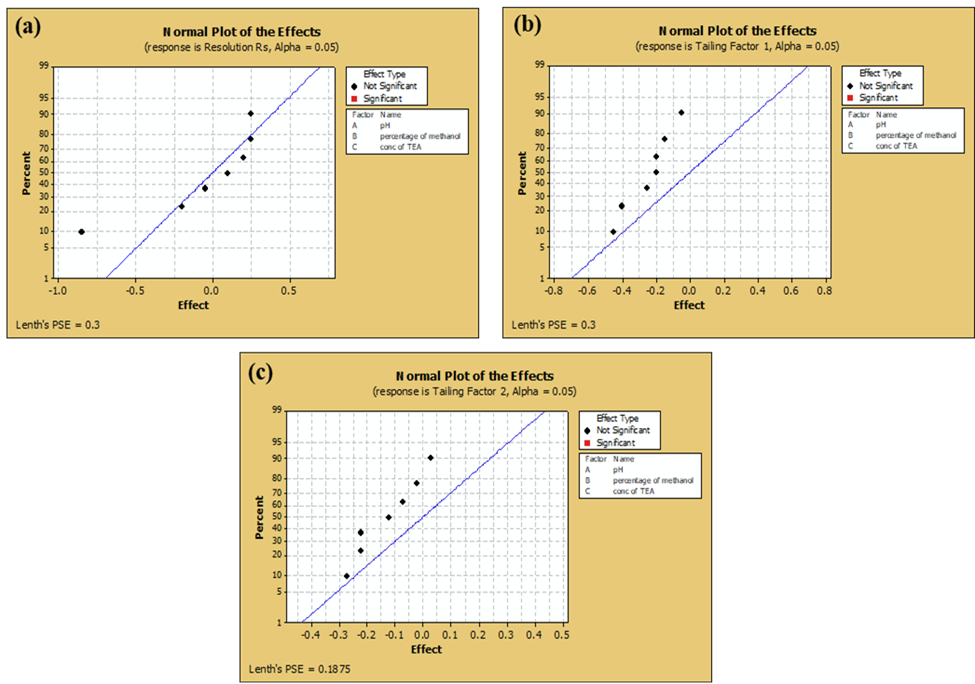


Fig. S3: 2^3^ full factorial design normal plots of the effects on the chromatographic responses at alpha = 0.05.


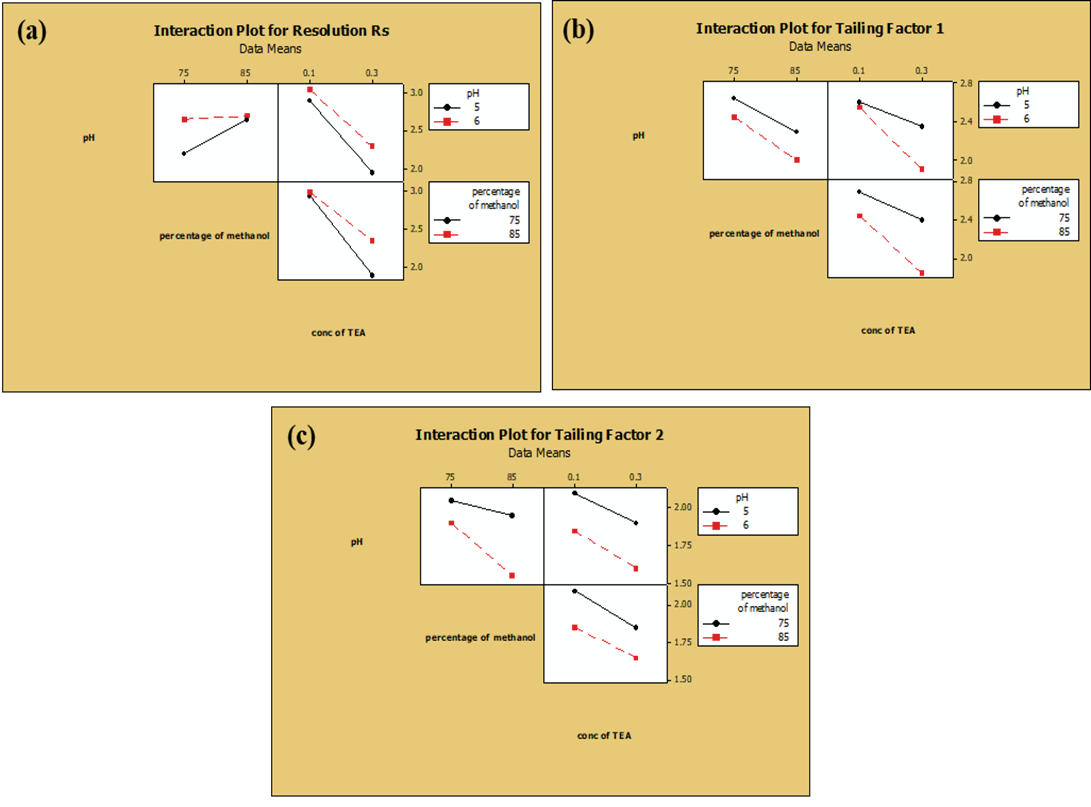


Fig. S4: 2^3^ full factorial design interaction plots for chromatographic responses by data means type.
